# Cyclophosphamide-associated partial proximal tubular dysfunction

**DOI:** 10.17179/excli2025-8622

**Published:** 2025-07-10

**Authors:** Rutvikkumar Jadvani, Angel Juarez, Chintan V. Shah

**Affiliations:** 1 Division of Nephrology, Hypertension, and Renal Transplantation, University of Florida College of Medicine, Gainesville, Florida, USA

## ⁯⁯

Fanconi syndrome is a disorder characterized by a global defect in the proximal tubule, leading to impaired reabsorption of various electrolytes by the proximal tubule (Keefe and Bokhari, 2023[1]). Although proximal tubular toxicity and dysfunction are well described with ifosfamide (Sprangers and Lapman, 2020[4]), we present, to the best of our knowledge, the first case of partial proximal tubular dysfunction (Fanconi-like syndrome) associated with cyclophosphamide. 

A 62-year-old female with a history of Ehlers-Danlos syndrome, congestive heart failure with preserved ejection fraction, and refractory neurosarcoidosis with previously failed therapies, including high-dose steroids, methotrexate, infliximab, azathioprine, but was well controlled after receiving high-dose cyclophosphamide 1,620 mg (750 mg/m^2^ × 2.16 m^2^) every 4 weeks for 13 months. The patient presented with diffuse muscle cramps and fatigue, within 2 months of the last dose of cyclophosphamide, without any history of vomiting or diarrhea. Laboratory results were remarkable for serum sodium 135 meq/L, potassium 2.2 meq/L, phosphate 1.7 mg/dL, uric acid 2.4 mg/dL, magnesium 1.9 mg/dL, total serum CO2 32 mmol/L, and serum creatinine of 1.1 mg/dL (97.2 µmol/L). She received aggressive oral and intravenous electrolyte supplementation. Although severe hypokalemia could be explained by the periodic use of loop and thiazide-like diuretics, the presence of severe hypouricemia and hypophosphatemia in the setting of a high-purine and phosphorus diet (daily consumption of carbonated beverages and animal protein) was suspicious for proximal tubular dysfunction. Cyclophosphamide was held, and the patient was established with an onco-nephrology service to confirm the diagnosis further.

The patient, as an outpatient, had their oral phosphorus supplements held, leading to a decline in serum phosphorus to 3.3 mg/dL, a 24-hour urine phosphorus level of 1232 mg/day, with a fractional excretion of phosphorus (FePO4) of 21%. After three weeks without supplements, hypophosphatemia worsened (serum phosphorus 2.7 mg/dL with FePO_4_ 11 %), confirming renal phosphorus wasting. The diagnosis was confirmed to be partial proximal tubular dysfunction (partial Fanconi syndrome) without glucosuria or metabolic acidosis. Oral potassium phosphorus 500 mg three times a day and potassium chloride 20 meq twice daily were resumed. Nine months after the last dose of cyclophosphamide, withdrawing phosphorus supplements for four weeks again caused hypophosphatemia (serum phosphorus 2.4 mg/dL with FePO_4_ 14.63 %), indicating persistent proximal tubular dysfunction (Supplementary information, Table S1). Urine amino acids quantitation by mass spectrometry (LC MS/MS) revealed high levels of alanine 710 µmol/g (normal 60-500 µmol/g), leucine 53 µmol/g (less than 45 µmol/g), taurine 10703 µmol/g (normal less than 3200 µmol/g), and tryptophan 101 µmol/g (15-95 µmol/g) amino acids in urine, suggesting selective aminoaciduria rather than massive, generalized aminoaciduria consistent with partial rather than complete Fanconi syndrome. Genetic work-up (Renasight^TM^, Natera Inc.) revealed a heterozygous mutation in Fanconi Anemia, Group F (FANCF) (c.241G>T (p.Ala81Ser), a genetically heterogeneous recessive disorder characterized by cytogenetic instability and hypersensitivity to DNA crosslinking agents (Léveillé et al., 2004[2]), such as cyclophosphamide. Cyclophosphamide was permanently discontinued, and the patient is being considered for rechallenge with infliximab.

Ifosfamide and cyclophosphamide, structural isomers, are known for their urotoxic side effects. Both inhibit thymidine incorporation in DNA and RNA synthesis, affecting the proximal tubule (Mohrmann et al., 1994[3]). While ifosfamide is strongly associated with Fanconi syndrome (Sprangers and Lapman, 2020[4]), cyclophosphamide has never been reported to cause proximal tubular dysfunction, primarily because it is not selectively transported into these cells (Sprangers and Lapman, 2020[4]; Mohrmann et al., 1994[3]). This report highlights that cyclophosphamide, at high doses over extended periods, can cause partial proximal tubular dysfunction, particularly in patients with undiagnosed genetic defects. Clinicians should maintain a high index of suspicion for this adverse effect.

## Conflict of interest

The authors have no conflict of interest to declare.

## Supplementary Material

Suppl.-information
