# Supplementary material for: Cyclophosphamide-associated partial proximal tubular dysfunction
Source: EXCLI J. 2025 Jul 10;24:777–8. doi: 10.17179/excli2025-8622 (PMC12381363; doi:10.17179/excli2025-8622)
Supplement: Suppl.-information [file EXCLI-24-777-s-001.pdf]

EXCLI Journal 2025;24:777-778 – ISSN 1611-2156
Received: June 03, 2025, accepted: June 17, 2025, published: July 10, 2025

Supplementary information to:

Letter to the editor:

CYCLOPHOSPHAMIDE-ASSOCIATED PARTIAL PROXIMAL TUBULAR DYSFUNCTION

Rutvikkumar Jadvani, Angel Juarez, Chintan V. Shah*

Division of Nephrology, Hypertension, and Renal Transplantation,
University of Florida College of Medicine, Gainesville, Florida, USA

* **Corresponding author:** Chintan V. Shah, MD, Division of Nephrology, Hypertension, and Renal Transplantation, University of Florida College of Medicine, 1600 SW Archer Road, Room CG-98, Gainesville, FL 32610, Office: (352) 294-8790;
E-mail: shahc@ufl.edu

https://dx.doi.org/10.17179/excli2025-8622

This is an Open Access article distributed under the terms of the Creative Commons Attribution License (https://creativecommons.org/licenses/by/4.0/).

EXCLI Journal 2025;24:777-778 – ISSN 1611-2156
Received: June 03, 2025, accepted: June 17, 2025, published: July 10, 2025

Table S1: Serum and urine chemistry

	**Serum Chemistry**	
On presentation	Sodium<br>Potassium<br>Phosphate<br>Uric acid<br>Magnesium<br>Total serum CO_2<br>Creatinine<br>PTH<br>25-OH, Vitamin D 1-25<br>dihydroxy vitamin D	135 meq/L<br>2.2 meq/L<br>1.7 mg/dL<br>2.4 mg/dL<br>1.9 mg/dL<br>32 mmol/L<br>1.1 mg/dL<br>68 pg/ml<br>32 ng/mL<br>39.7 pg/mL
	**Serum phosphorus level**	**Fractional excretion of phosphorus ($FePO_4$)**
On presentation	3.3 mg/dL	21 %
At 3 months follow-up[a]	2.7 mg/dL	11 %
At 9 months follow-up[a]	2.4 mg/dL	14.63 %
	**Urine Amino Acids**	
		Reference Range
At 9 months follow-up	Alanine 710 µmol/g<br>Leucine 53 µmol/g<br>Taurine 10703 µmol/g<br>Tryptophan 101 µmol/g	60-500 µmol/g<br>< 45 µmol/g<br>< 3200 µmol/g<br>15-95 µmol/g

[a] Serum phosphorus levels were measured after holding oral phosphorus supplements for 4 weeks.
